# New graduates’ perceptions of preparedness to provide speech-language therapy services in general and dysphagia services in particular

**DOI:** 10.4102/sajcd.v62i1.110

**Published:** 2015-06-18

**Authors:** Shajila Singh, Alannah Booth, Fadziso Choto, Jessica Gotlieb, Rebecca Robertson, Gabriella Morris, Nicola Stockley, Katya Mauff

**Affiliations:** 1 Department of Health and Rehabilitation Sciences, University of Cape Town, South Africa; 2 Speech and Language Therapist, Julie A Cardona and Associates, South Africa; 3 Speech-Language Therapist, Tintswalo Hospital, Acornhoek, Mpumalanga, South Africa; 4 Speech-Language Therapist, Sandton Therapy Suites, South Africa; 5 Speech-Language Therapist, Olivia Thomet and Associates, South Africa; 6 Speech-Language Therapist, Themba Hospital, Mpumalanga, South Africa; 7 Speech-Language Therapist, Luanet Smit practice, South Africa; 8 Department of Statistical Sciences, University of Cape Town, South Africa

## Abstract

**Background:**

Upon graduation, newly qualified speech-language therapists are expected to provide services independently. This study describes new graduates’ perceptions of their preparedness to provide services across the scope of the profession and explores associations between perceptions of dysphagia theory and clinical learning curricula with preparedness for adult and paediatric dysphagia service delivery.

**Methods:**

New graduates of six South African universities were recruited to participate in a survey by completing an electronic questionnaire exploring their perceptions of the dysphagia curricula and their preparedness to practise across the scope of the profession of speech-language therapy.

**Results:**

Eighty graduates participated in the study yielding a response rate of 63.49%. Participants perceived themselves to be well prepared in some areas (e.g. child language: 100%; articulation and phonology: 97.26%), but less prepared in other areas (e.g. adult dysphagia: 50.70%; paediatric dysarthria: 46.58%; paediatric dysphagia: 38.36%) and most unprepared to provide services requiring sign language (23.61%) and African languages (20.55%). There was a significant relationship between perceptions of adequate theory and clinical learning opportunities with assessment and management of dysphagia and perceptions of preparedness to provide dysphagia services.

**Conclusion:**

There is a need for review of existing curricula and consideration of developing a standard speech-language therapy curriculum across universities, particularly in service provision to a multilingual population, and in both the theory and clinical learning of the assessment and management of adult and paediatric dysphagia, to better equip graduates for practice.

## Introduction

Frenk et al. (2010) suggest that health professional education has not kept pace with the challenges facing health systems. In particular, it was suggested that curricula have produced graduates who are ill-equipped to address the needs of patients and populations. South African studies in speech-language therapy (Modi & Ross, 2000; Penn, Mupawose & Stein, 2009; Wranz, 2011) reflect this trend, finding that graduates were not sufficiently prepared by some university curricula to provide dysphagia services.

Graduates’ views have been used as one way of assessing curriculum quality. Modi and Ross's (2000) survey of 50 hospital based speech-language therapists (SLTs) revealed gaps in dysphagia knowledge and skills, which participants linked to the quality of their training programme: that is either the lack of a (stand-alone) dysphagia course or to poorly structured courses with insufficient information offered. There was also a lack of clinical training in dysphagia, leading to a rating of education in dysphagia as poor. Penn et al. (2009), in describing the community service experiences of 132 graduates from the University of the Witwatersrand, reported that many felt unprepared to provide dysphagia services, with a further challenge related to dealing with the linguistic diversity of patients. Similarly, Wranz (2011) reported on the community service experiences of 18 graduates from Stellenbosch University. Less than half of these graduates (41% of the paediatric population and only 30% of those who treated adults) felt confident to provide services in dysphagia and recommended that more clinical practice in dysphagia be incorporated into training programmes. Other areas of the curriculum perceived to be less than optimal included cerebral palsy, sign language, craniofacial abnormalities (Wranz, 2011) and service delivery relating to the linguistic and cultural diversity of patients. Concerns about new graduates’ lack of preparedness have also been expressed by representatives of public health sector SLTs, (who work with new graduates in compulsory community service) at the annual meetings of the National Speech Therapy and Audiology (STA) Forum. This forum recommended that university curricula provide students with more clinical learning opportunities with patients with autism, cleft lip and palate, cerebral palsy, severe disabilities, traumatic brain injuries, adult and paediatric dysphagia, paediatric apraxia and coping in a multilingual environment (National STA Forum, 2012, 2013).

It may well be true that new graduates lack confidence, which reflects their novice status, and in some countries such as the USA, Ireland (IASLT, 2012) and New Zealand (New Zealand Speech-Language Therapists Association, 2015), there is a period of supervised practice following graduation. In South Africa, all new speech-language therapy graduates are required to serve a year of compulsory community service in the public health sector hospitals and clinics, where they may not be supervised by a SLT (Penn et al., 2009; Wranz, 2011). There are a limited number of SLTs employed in the public health sector and community service graduates are placed in areas where there have previously been no speech-language therapy services, in an attempt to increase access to such services (Harrison, 2009). The Health Professions Council of South Africa Regulations for the undergraduate curricula and professional examinations in speech language therapy (HPCSA, 2012) stipulate that new graduates must be able to practise independently.

It has been more than a decade since the Modi and Ross (2000) study and later research has reported on graduates from only two of the six universities that educate and train SLTs in South Africa. The current study set out to explore the perceptions of preparedness of new graduates, from all six universities, to provide speech-language therapy services in general and dysphagia services in particular. The results of this study could be used to inform the speech-language therapy and dysphagia curricula offered at South African universities

The aims of this study were: (1) to describe SLT graduates’ perceptions of preparedness to provide speech, language and communication services across the scope of the speech language therapy profession and paediatric and adult dysphagia services and (2) to explore associations between perceptions of dysphagia theory and clinical learning curricula with preparedness for dysphagia service delivery.

## Methodology

There were 129 (speech-language therapy and speech-language therapy and audiology) graduates who met the inclusion criteria of having graduated in 2011 and being employed as community service SLTs in 2012. Total population sampling (Lund Research Ltd, 2010) resulted in all graduates who were contactable (*N* = 126) being included. Within the first three months of the year, 80 graduates participated in the study, yielding a response rate of 63.49%. The response rate of graduates at the different universities ranged from 52% to 76% (Table 1). Although more participants from universities 3 and 5 were placed at urban community service sites, the overall distribution of placements was 53.75% urban and 46.25% rural. There were 55% who were registered as speech-language therapists and audiologists and 45% as SLTs. Whilst 84% (*n* = 67) reported being supervised at their community service site, 16% (*n* = 13) had no supervision and 21% (*n* = 17) were not supervised by an SLT. Eighty-two percent (*N* = 73) reported assessing and managing a patient with dysphagia at their community service site. Seven (8.75%) participants did not complete the entire questionnaire.

**TABLE 1:** Participants.

University	Total N at university	Number of participants	% response rate	Placement		Registration		Supervision at community service site	
				Rural	Urban	SLT	SLT/A	No	Yes	SLT
1	14	10	71	9	1	-	10	6	4	4
2	17	13	76	6	7	13	-	-	13	9
3	38	20	53	5	15	4	16	2	18	16
4	21	11	52	7	4	11	-	3	8	5
5	25	18	72	2	16	-	18	2	16	14
6	14	8	57	8	0	8	-	-	8	2
**Total**	**129**	**80**	**-**	**37**	**43**	**36**	**44**	**13**	**67**	**50**
	**-**	**62**%	**-**	**46**%	**54**%	**45**%	**55**%	**16**%	**84**%	**63**%

Note: Registration (with the HPCSA): SLT, Registered as a speech-language therapist; SLT/A, Registered as a speech-language therapist/audiologist.

### Questionnaire

An electronic, self-administered questionnaire (SurveyMonkey™) was designed to obtain information on: (1) participants’ demographics including university attended, type of qualification, details of the community service placement site, and availability and nature of supervision, (2) perceptions of preparedness to provide services across the scope of the SLT profession (Wranz, 2011), including fluency, voice, articulation and phonology, child and adult apraxia, dysarthria and dysphagia, early communication intervention, child language, literacy, paediatric aural rehabilitation, aphasia, traumatic brain injury, cognitive communication and augmentative and alternative communication. Preparedness to provide services in African languages and in sign language was also included.

The questionnaire was also designed to obtain information on (3) the theory curricula (Modi & Ross, 2000; Wranz, 2011) and on (4) the clinical learning curricula, in paediatric and adult dysphagia, and (5) the perceptions of the adequacy of the curricula as preparation to provide dysphagia services. The section on the theory probed whether there was such a course, the number of hours allocated to it, coverage of the basic sciences linked to dysphagia (i.e. anatomy, physiology, neurology and pathology of swallowing), the (clinical and objective) assessment and the management of dysphagia. The clinical education section required information on clinical learning opportunities in dysphagia including clinical and objective assessments and management of cases.

Face validity (Burns et al., 2008) was addressed by having SLT professionals who had clinical experience and who had conducted postgraduate research in the field of paediatric and adult dysphagia and two graduates (class of 2008 and 2010) provide feedback on the questionnaire. The questionnaire was modified based on the feedback received.

Reliability of the questionnaire was addressed by varying response formats to limit a response set (Siniscalco & Auriat, 2005). Closed-response formats included checklists (Kanjee, 2006), positively and negatively worded items (Kaplan & Saccuzzo, 2008) on a four point Likert scale without a neutral option (Finchilescu, 2002) and dichotomous items (Kanjee, 2006). Open response formats were included to allow participants to elaborate and expand on questions answered previously and to provide alternative responses (Siniscalco & Auriat, 2005).

### Procedures

The University of Cape Town's Faculty of Health Sciences’ Human Research Ethics Committee approved the study (HREC REF 553/2011). A pilot study was conducted with two graduates who reviewed the questionnaire and reported on its flow, ease, length, wording and relevance of items, as well as the layout and response formats. The pilot also addressed face validity (Lodico, Spaulding & Voegtle, 2010) and resulted in changes being made to the questionnaire to improve the clarity of the questions and layout.

In the main study, each of the 2011 graduates for whom contact details were available (*N* = 126) were sent a text message (Fan & Yan, 2010) alerting them to expect a telephone call. They were telephonically informed about the purpose and nature of the study and email addresses were obtained following indications of willingness to participate. The email contained the study information letter and consent form, a link to the electronic questionnaire and the researchers’ contact details. Participants signalled consent by completing the questionnaire. An email reminder was sent out 10 days later (Burns et al., 2008).

### Data analysis

Since the researchers were students and faculty at one of the universities, single blinding was used to eliminate researcher bias (Peck & Devore, 2010), as well as to maintain the anonymity of university affiliation. Only two researchers had access to the details of university affiliation. They assigned a code to each institution rendering the other researchers blind to this information. These two researchers did not participate in the data capture and analysis.

Responses on the four point Likert scale were collapsed with strongly agree and agree constituting ‘agree’ and strongly disagree and disagree constituting ‘disagree’. Some participants did not answer all of the questions, resulting in missing data. The results were based on the analysis of all cases where data was available. Descriptive and inferential, non-parametric correlation statistical analyses were conducted using Stata (version 11). Fisher's Exact Test was used to calculate the exact probability (Boslaugh & Watters, 2008) and to identify whether there was a significant relationship between variables; correspondence analysis biplots were used to identify the strongest association between the variables (Greenacre, 2010). The Kruskal-Wallis rank test was corrected for tied ranks and used to compare continuous data with more than two samples that were independent (Corder & Foreman, 2009). Categories in which there was only one observation were excluded when conducting the Kruskal-Wallis test. The alpha level was set at 0.05, with Bonferroni correction to adjust the comparative *p* value following multiple independent tests of significance to decrease the chances of a Type I error (Hayes, 2005).

## Results

**FIGURE 1:**
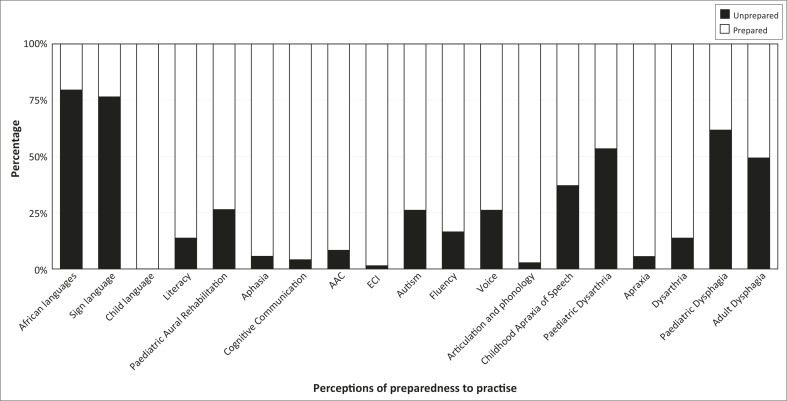
New graduates’ perceptions of preparedness to provide SLT services (*N* = 73).

Participants’ perceptions of preparedness to provide SLT services varied across the scope of professional practice (Figure 1). Generally, participants perceived themselves to be well prepared to provide services in a range of areas, including child language (100%), early communication intervention (98.6%) and articulation and phonology (97.26%). A quarter to a third of participants felt unprepared to provide services in autism (26.03%), paediatric aural rehabilitation (26.39%), voice (26.03%) and childhood apraxia of speech (37%). Approximately half felt unprepared to provide services in adult dysphagia (49.3%), paediatric dysarthria (53.42%) and paediatric dysphagia (61.64%). Participants experienced the greatest degree of unpreparedness to provide services requiring sign language (76.39%) and African languages (79.45%).

### Perceptions of preparedness to practice in dysphagia across universities

**FIGURE 2:**
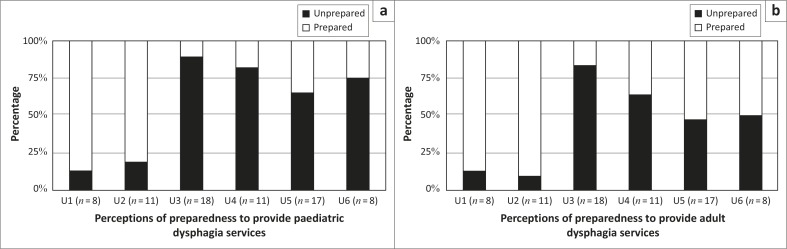
Perceptions of preparedness to provide paediatric and adult dysphagia services across universities (*N* = 73).

Perceptions of preparedness to provide dysphagia services varied across universities (Figure 2). In paediatric dysphagia, preparedness ranged from 11% to 88% with the majority of participants from four universities (i.e. University 3, 4, 5 and 6) feeling unprepared. Preparedness to provide adult dysphagia services varied from 17% to 91% across universities, with most participants at three universities (i.e. University 3, 4 and 6) feeling unprepared.

### Dysphagia curricula

Universities offered theory and clinical learning in adult and paediatric dysphagia in either the third or fourth year of study (Table 2). Perceptions of the median time for paediatric and adult theory was reported to range from 4 to 48 hours and from 12 to 59 hours respectively; for clinical learning it ranged from 5 to 30 hours and 3.5 to 40 hours respectively. Some participants reported no clinical learning with paediatric (*n* = 20; 25.6%) and adult (*n* = 14; 16.1%) dysphagia.

**TABLE 2:** Perceptions of dysphagia curricula time and preparedness to practise.

University	Theory						Clinical learning					
	Year of study	Median time		Sufficient time			Year of study	Median time			Sufficient time			Prepared to practice		
		N	Hours	N	n	%		N	n	Hours	N	n	%	N	n	%
**Paediatric Dysphagia**							
1	3	10	25.00	10	8	80.0	4	9	-	30.0	9	5	55.6	8	7	87.5
2	3	13	48.00	13	11	84.6	4	13	-	18.0	13	5	38.5	11	9	81.8
3	3	20	20.00	20	5	25.0	3	20	9	5.0	19	1	5.3	18	2	11.1
4	4	9	19.00	11	1	9.1	4	11	5	25.0	11	2	18.2	11	1	9.0
5	3	18	14.75	17	6	35.3	4	17	-	20.0	17	6	35.3	17	6	35.3
6	4	6	4.00	8	1	12.5	4	8	6	14.5	8	0	0.0	8	2	25.0
**Adult dysphagia**							
1	3	10	47.5	10	8	80.0	4	9	-	36	9	7	77.7	8	7	87.5
2	3	13	59.0	13	10	76.9	3.4	12	-	40	13	9	69.2	11	10	90.9
3	3	20	20.0	20	6	30.0	3	20	5	8	19	1	5.3	18	3	16.6
4	4	11	40.0	11	3	27.2	4	11	9	3.5	11	0	0.0	11	4	36.3
5	3	17	12.0	17	10	58.8	4	20	-	20	17	7	41.2	17	9	52.9
6	4	8	17.5	8	2	25.0	4	15	-	15	8	1	12.5	8	4	50.0

*N*, number of participants responding to the question; *n*, no training.

**FIGURE 3:**
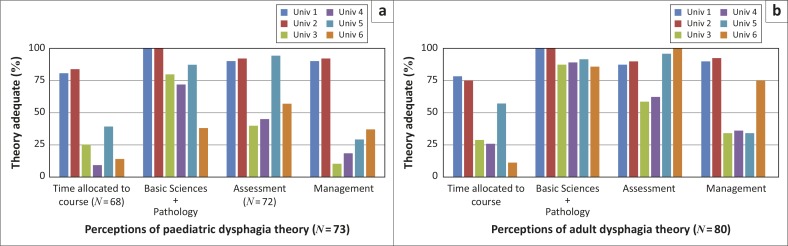
Perceptions of the adequacy of the dysphagia theory curricula across universities.

**FIGURE 4:**
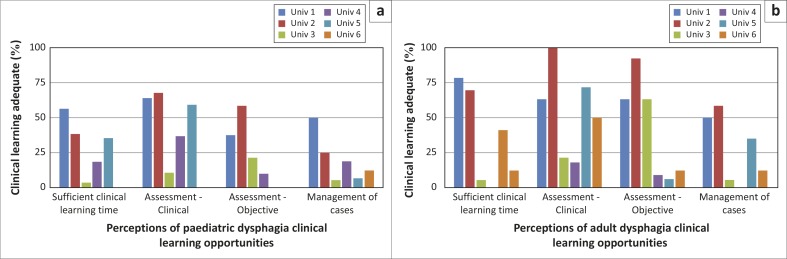
Perceptions of the adequacy of the clinical learning in dysphagia across universities.

Perceptions of the theory and the clinical learning curricula varied at the different universities (Figure 3 and Figure 4 respectively). Basic sciences, which included anatomy, physiology, neurology and pathology, were perceived to have been addressed relatively comprehensively by five universities. The theory relating to the assessment of paediatric dysphagia was perceived to have been adequate at only three universities with better coverage for adults. Theory relating to the management of dysphagia was sufficiently addressed at only two universities for children and at three for adults.

Participants’ perceptions were of limited clinical learning opportunities (Figure 4), particularly marked in the objective assessment and in the management of both paediatric and adult dysphagia. Some universities reportedly offered no or very limited clinical learning.

There were significant associations between elements of the theory and clinical learning curricula and perceptions of preparedness to practice (Table 3).

**TABLE 3:** Association between elements of the theory and clinical learning curricula and perceptions of preparedness for dysphagia practice.

Variable	Paediatric dysphagia			Adult dysphagia		
	Association with preparedness to practice		Correspondence analysis	Association with preparedness to practice		Correspondence analysis
	-	*p*		-	*p*	
**Theory**						
Curriculum time	*χ*^2^(2, *N* = 68) = 7.39	0.025	Higher hours associated with preparedness	*χ*^2^(3, *N* = 73) = 8.024	0.046	Higher hours associated with preparedness
Basic sciences (anatomy, neurology, physiology, pathology)	*c*^2^(9, *N* = 73) = 11.37	0.154	Adequate knowledge not associated with preparedness	*c*^2^(6, *N* = 73) = 16.89	0.004	Inadequate knowledge associated with unpreparedness
Normal development of swallowing	*χ*^2^(9, *N* = 73) = 15.57	0.057	Adequate knowledge not associated with preparedness	-	-	-
Assessment	*c*^2^(9, *N* = 72) = 15.96	0.030	Inadequate knowledge associated with unpreparedness	*c*^2^(9, *N* = 73) = 27.18	0.001	Inadequate knowledge associated with unpreparedness
Management	*χ*^2^(9, *N* = 73) = 31.59	0.000	Inadequate knowledge associated with unpreparedness	*c*^2^(9, *N* = 73) = 43.59	0.000	Inadequate knowledge associated with unpreparedness
**Clinical learning**						
Clinical learning time	*χ*^2^(9, *N* = 73) = 26.19	0.004	Adequate time associated with preparedness	*χ*^2^(9, *N*= 73) = 35.54	0.000	Inadequate time associated with unpreparedness
Assessment – clinical	*χ*^2^(9, *N* = 73) = 19.33	0.005	Lack of learning associated with unpreparedness	*χ*^2^(9, *N* = 73) = 52.62	0.000	Lack of learning associated with unpreparedness
Assessment – objective	*χ*^2^(9, *N* = 73) = 44.95	0.022	Lack of learning associated with unpreparedness	*χ*^2^(9, *N* = 73) = 32.76	0.001	Adequate learning associated with preparedness
Management of a range of cases	*χ*^2^(9, *N* = 73) = 34.98	0.000	Lack of learning associated with unpreparedness	*χ*^2^(9, *N* = 73) = 52.98	0.000	Lack of learning associated with unpreparedness

These results demonstrate that perceptions of inadequate theoretical coverage and lack of clinical learning opportunities with the assessment and the management of both paediatric and adult dysphagia were significantly associated with poor preparedness for practice.

## Discussion

There are several areas of speech-language therapy professional practice in which graduates from all universities perceived being well prepared to offer services including child language, early communication intervention, articulation and phonology, aphasia, cognitive communication disorders, apraxia and augmentative and alternative communication. The high levels of perceived preparedness offer reassurance about the quality of SLT training in these areas in South Africa.

Less reassuring are findings suggesting that approximately a quarter of participants felt unprepared in the areas of autism, paediatric aural rehabilitation, voice and childhood apraxia of speech. A bigger challenge for new graduates appeared to be in the areas of paediatric dysarthria and adult and paediatric dysphagia: approximately 50% perceived themselves to be unprepared. The areas of practice that caused the greatest feelings of a lack of preparedness related to providing services in sign and African languages. These results echo those of earlier studies (Modi & Ross, 2000; Wranz, 2011) and the concerns of the National Speech Therapy Audiology Forum (National STA Forum, 2012, 2013) and suggest a need for urgent review of speech-language therapy curricula by institutions in order to graduate practitioners who feel better prepared to offer services across the professional scope of practice in South Africa.

In disaggregating the results pertaining to perceptions of preparedness to offer dysphagia services, there was variability across institutions, with the graduates of only two institutions perceiving themselves to be prepared to provide adult and paediatric dysphagia services. Exploration of perceptions of the adult and paediatric dysphagia curricula suggests that graduates from the different universities have had different learning experiences and coverage of particular areas. Theoretical coverage of basic sciences (i.e. anatomy, physiology, neurology and pathology) was perceived to have been addressed adequately by most institutions but was not sufficient for graduates to feel prepared to practise. Not surprisingly, perceptions of greater preparedness were consistently linked to perceptions of more curriculum space being devoted to the theory course with better theoretical coverage of assessment and management of dysphagia. Sufficient clinical learning time and opportunities for practice with clinical and objective assessments and management of an adequate number of cases were also linked to perceptions of greater preparedness. Such findings are consistent with studies suggesting that low ratings for preparedness are linked to low ratings for ‘depth and/or breadth or number of learning opportunities’ (Rose, McAlpine & Strychar, 2005), that graduates were most confident in managing cases to which they had been frequently exposed during their training (Arena, Kruger, Holley, Millar & Tenant, 2007) and that there was a desire for more rigorous clinical education (Hart & Macnee, 2007; Woods et al., 2014).

Recommendations include the adoption of a standard theoretical and clinical curriculum in adult and paediatric dysphagia. Such a curriculum should provide students with sufficient learning opportunities to acquire the theory and clinical learning experiences with the assessment and management of dysphagia across the lifespan. The Board for Speech Language and Hearing Professions of the HPCSA, in its regulations, specifies the exit level outcomes that undergraduates must meet to practise safely and independently as new SLTs. However, in light of the non-uniformity of graduates’ preparedness, it is evident that there are differences in training standards despite the regulations. Thus, the Board may wish to consider explicitly setting the standard for the dysphagia curricula to ensure that similar learning opportunities are afforded to all students in all training programmes to better prepare them for practice. A national Board examination, which all final year students take, may also assist with objectively assessing the levels of graduates’ competence and performance or preparedness to practice and indirectly reflect the quality and uniformity of learning opportunities. Dysphagia may be seen to be a relatively new area of professional practice and there may therefore be a need for ‘training of the trainers’ in the areas of adult and paediatric dysphagia as these may not have been part of the educators’ training.

Implications of the lack of preparedness of graduates to offer dysphagia services are not known. Some practitioners may take additional courses or CPD activities in the areas in which they feel the need or in which they frequently practise to develop their knowledge and skills. In instances where SLTs offer services when they feel unprepared, the outcomes for patients may be less than optimal. Some SLTs may avoid treating cases they feel unprepared to assess and manage, thereby reducing access to much-needed services. The consequences of SLTs’ perceived lack of preparedness needs further research to explore the impact.

There may be a need for CPD or short courses to assist practitioners to acquire the necessary competence, knowledge and skills required to provide such services, so as not to disadvantage them and their patients who require such services. Blumenthal, Gokhale, Campbell and Weissman (2001) suggest that focused short-term training can remedy some gaps in physicians’ skills, but that such approaches are inherently less desirable than providing adequate preparation as a part of routine training. In a similar vein, training in dysphagia may be better addressed within the undergraduate curricula than ad hoc CPD courses later on.

In a country in which there are 11 official languages, it is perhaps not surprising that 79% of new graduates felt unprepared to offer services in African languages. It is of concern, however, because of the impact this lack of preparedness may have on service provision to the majority (75.2%, according to African Languages and Literature (2009); note that this number does not include the speakers of Afrikaans) of the South African population who speak an African language as their first language. Perry (2012) suggests that where there are gaps in cultural and linguistic competency, practitioners may develop negative perceptions and attitudes towards clients, which interfere with effective clinical service delivery and reduce the effectiveness of outcomes for patients. Speech-language therapy, as a profession, is quintessentially a profession that addresses clients’ concerns about their speech and language comprehension and production – in the clients’ first language. An SLT's lack of preparedness to cope with linguistic diversity renders attempts to provide language-based interventions sub-optimal. The SLT profession in the country needs to urgently consider how to optimise service provision in the context of many different languages of patients, including but not limited to: conducting research that facilitates cross-linguistic service provision, developing assessment and management tools in different languages and recruiting, training and graduating a greater number of individuals who speak African languages; there is also a need to skill SLT students to work cross linguistically, optimise service delivery with bilingual clients, train and work with interpreters and to acquire skills to learn to speak at least one African language that is relevant to the local context, in order to be able to provide therapy for speech and language disorders in that language. Without some intervention, the profession faces the risk of not providing optimal SLT management for the majority of the population.

Frenk *et al* (2010) suggest that curricula in the new century should prepare graduates to be relevant and better matched to patient and population needs. Whilst it may be difficult to train students to equal competence in all areas of practice, perhaps the burden of disease or profile of SLT cases in the public and private health and education sector should be considered, with exit competencies mirroring this profile. Preliminary results of a study (work in progress) by the first author suggest that dysphagia is the most frequently occurring condition at most South African public sector hospitals where SLTs work. It is important to improve access and standards of dysphagia service delivery across the country. SLT training institutions will need to grapple with the challenges inherent in preparing graduates for practice within the South African context and adapt their curricula accordingly.

### Limitations

This study is limited by reporting on new graduates’ perceptions, which may be different from actual tested preparedness for practice and which is subject to biases associated with memory and recall (Lieberman & Hilliard, 2006). New graduates may not be able to judge how well they were trained if they have not encountered problems or particular patient groups (Lieberman & Hilliard, 2006). Nevertheless, this study does provide some valuable insights into new graduates’ preparedness to provide speech-language therapy services in the public health sector. In addition, studies have shown that self-reported high levels of preparedness are correlated with good performance (Shubert, Tetzlaff, Tan, Ryckman & Mascha, 1999) and may be used as an indicator of the quality of education (Cantor, Baker & Hughes, 1993). Ratings of the quality of instruction have been related to perceptions of preparedness to provide treatment. An enhanced curriculum increased perceptions of preparedness to diagnose and treat and educational quality ratings (Wakeman, Pham-Kanter, Baggett & Campbell, 2014). Further research is required on the actual content and quality of the curricula offered in the different areas across universities.

## Conclusion

Education and training of speech-language therapists in South Africa is not uniform. There are areas of practice, including articulation and phonology and early communication intervention, in which most graduates, across institutions, feel prepared to offer SLT services. However, new graduates feel relatively less well prepared in a number of other areas of practice, most notably in dysphagia and provision of services in sign language and African languages. Perceptions of limited theoretical coverage and exposure to clinical assessment and management opportunities were associated with feelings of unpreparedness. Differences in university theory and clinical curricula, with differential coverage of, for example, assessment and management of adult and paediatric dysphagia, as perceived by new graduates, whilst not a reflection of actual preparedness, nevertheless suggest a need for review of existing curricula. Consideration may be given to developing a standard adult and paediatric dysphagia theory and clinical curriculum, national examinations and training of educators to improve graduates’ preparedness. Strategies to address skilling related to cross-linguistic service delivery for South African languages must be integrated into education and training. Qualified practitioners who feel unprepared will need to engage with continuing professional development to better capacitate themselves, to improve access and ensure quality service delivery.
